# Ultrasound characteristics of follicular and parafollicular thyroid neoplasms: diagnostic performance of artificial neural network

**DOI:** 10.1186/s13044-023-00168-2

**Published:** 2023-08-28

**Authors:** Michael Cordes, Theresa Ida Götz, Stephan Coerper, Torsten Kuwert, Christian Schmidkonz

**Affiliations:** 1 Radiologisch-Nuklearmedizinisches Zentrum, Nürnberg, Germany; 2 Department of Industrial Engineering and Health, Institute of Medical Engineering, Technical University Amberg-Weiden, Weiden, Germany; 3 Klinik für Allgemein und Viszeralchirurgie, Krankenhaus Martha-Maria, Nürnberg, Germany; 4 Clinic of Nuclear Medicine, University Hospital Erlangen, Erlangen, Germany

**Keywords:** Follicular thyroid neoplasms, Parafollicular thyroid neoplasms, Ultrasound, Artificial neural network

## Abstract

**Background:**

Ultrasound is the first-line imaging modality for detection and classification of thyroid nodules. Certain features observable by ultrasound have recently been equated with potential malignancy. This retrospective cohort study was conducted to test the hypothesis that radiomics of the four categorical divisions (medullary [MTC], papillary [PTC], or follicular [FTC] carcinoma and follicular thyroid adenoma [FTA]) demonstrate distinctive sonographic characteristics. Using an artificial neural network model for proof of concept, these sonographic features served as input.

**Methods:**

A total of 148 patients were enrolled for study, all with confirmed thyroid pathology in one of the four named categories. Preoperative ultrasound profiles were obtained via standardized protocols. The neural network consisted of seven input neurons; three hidden layers with 50, 250, and 100 neurons, respectively; and one output layer.

**Results:**

Radiomics of contour, structure, and calcifications differed significantly according to nodule type (*p* = 0.025, *p* = 0.032, and *p* = 0.0002, respectively). Levels of accuracy shown by artificial neural network analysis in discriminating among categories ranged from 0.59 to 0.98 (95% confidence interval [CI]: 0.57–0.99), with positive and negative predictive ranges of 0.41–0.99 and 0.78–0.97, respectively.

**Conclusions:**

Our data indicate that some MTCs, PTCs, FTCs, and FTAs have distinctive sonographic characteristics. However, a significant overlap of these characteristics may impede an explicit classification. Further prospective investigations involving larger patient and nodule numbers and multicenter access should be pursued to determine if neural networks of this sort are beneficial, helping to classify neoplasms of the thyroid gland.

## Background

Thyroid nodules are common in the general population. A recent update on related diagnostics indicates that ultrasound characteristics may be helpful in distinguishing benign from malignant growths [1]. To date, a number of researchers have detailed the ultrasound (US) features of thyroid carcinoma (medullary [MTC], papillary [PTC], or follicular [FTC]) and thyroid adenomas [2–5].

In a study by Kim et al., > 90% of MTCs proved to be solid in appearance, with ~ 50% harboring calcifications on US examinations [6]. Thus, MTCs did not differ substantially from PTCs in this regard. Fang et al. have also compared US features of PTCs with those of benign thyroid nodules [3]. Some US parameters (i.e., irregular shape, ill-defined margins, taller-than-wide [TTW] shape, and calcifications) were typically displayed by PTCs, distinguishing them from a heterogeneous group of benign nodules.

Yu et al. have examined US appearances of FTCs relative to follicular thyroid adenomas (FTAs) [7]. Echogenicity was similar in nature, but cystic elements were less often observed in FTCs than in FTAs. In a recent US investigation of ours, FTCs were also of significantly greater size, compared with PTCs or benign nodules [4].

The medical literature presently offers few comparative US analyses in which three distinct variants of thyroid carcinoma (MTC, PTC, FTC) and FTAs are addressed. US imaging is considered the gold standard for morphologic assessments of thyroid nodules and is generally recommended for those nodules detected clinically or through other imaging modalities [1]. Consequently, the American College of Radiology has devised the Thyroid Imaging Reporting and Data System (TI-RADS) as a standardized risk stratification system [8], necessitating some modifications over time [9].

In clinical practice fine-needle aspiration biopsies (FNABs) are performed for the evaluation of a benign or malignant nature of thyroid nodules. The clinical experience of the physician together with the US examination findings and a risk stratification system (i.e., TI-RADS), thyroid nodules will be selected for FNABs. However, some nodules might not be accessible for FNABs due to their anatomic localisation or due to the rejection of an FNAB by the patient. In these cases innovative artificial intelligence (AI) procedures might assist the physician in the assessment of thyroid nodules.

The diagnostic utility of a convolutional neural network (CNN) has recently been tested in a large series of patients with thyroid cancer [10]. During a retrospective and multicohort diagnostic study, based on US images, a CNN model (vs. skilled radiologists) showed similar sensitivity but improved specificity in identifying patients with thyroid cancer. Another CNN analysis reported even earlier involved TI-RADS classification of thyroid nodules [11]. The resultant performance in an open-access database was excellent (98.29%).

For the present proof-of-concept investigation, we used an artificial neural network model to test the hypothesis that MTCs, PTCs, FTCs, and FTAs have distinctive US features. All parameters selected for analysis were identified by cervical US studies.

## Materials and methods

This retrospective cohort study adhered to principles of the Declaration of Helsinki and its subsequent amendments. It also conformed to guidelines of the Institutional Review Board (IRB) of the Friedrich-Alexander-University, Erlangen/Nuremberg, Germany under auspices of the Bavarian Hospital Act (Bayerisches Krankenhausgesetz Art. 27 [4]). All subjects granted general permission for scientific use of their clinical data, supplying written informed consent for anonymous data publication. Ethics committee approval was waived since this was a retrospective study.

**Fig. 1:**
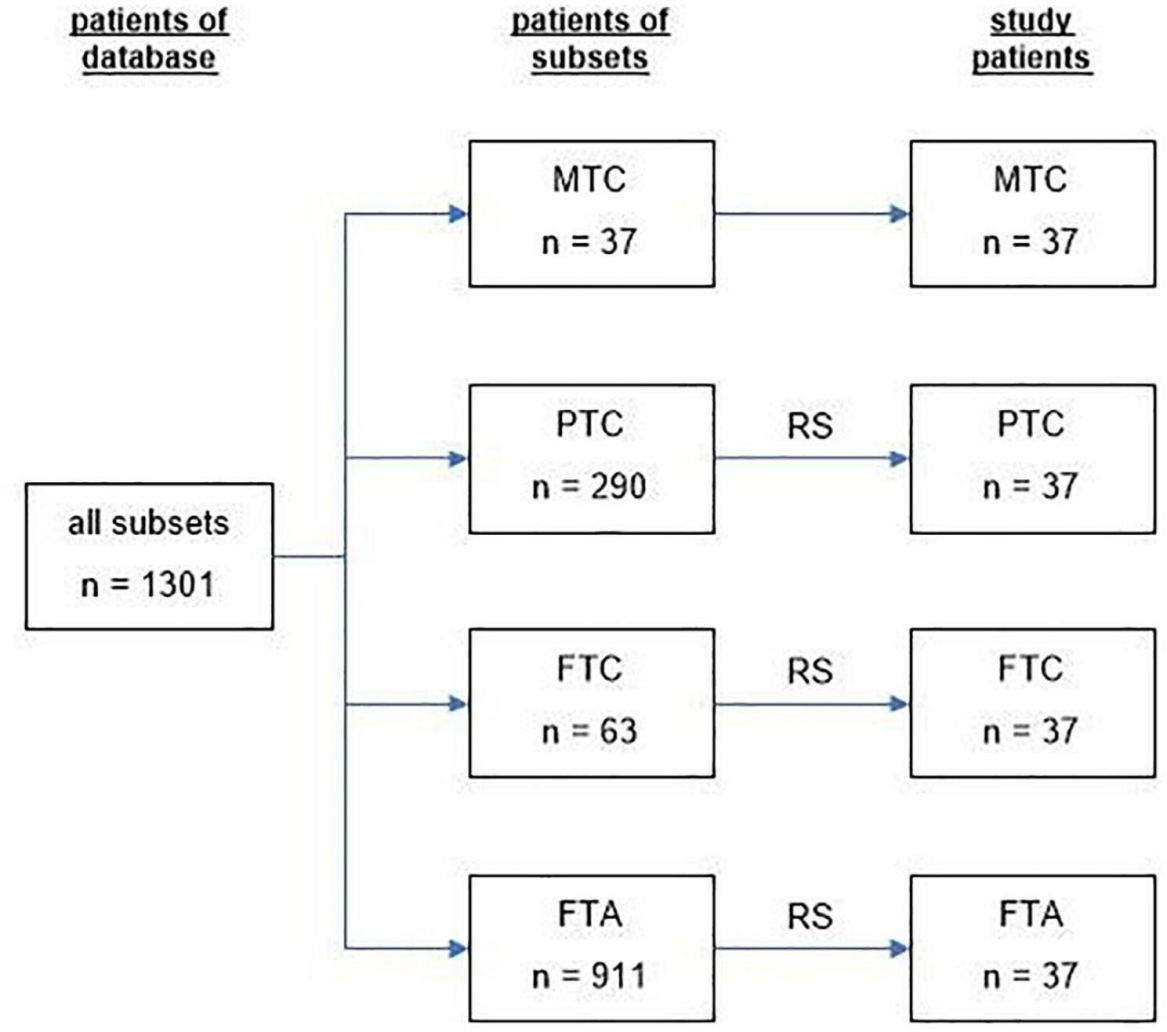
Recruitment algorithm of the study subjects. MTC: medullary thyroid carcinoma, PTC: papillary thyroid carcinoma, FTC: follicular thyroid carcinoma, FTA: follicular thyroid adenoma

We enrolled 148 patients (men, 43; women, 105) surgically treated for thyroid nodules between 2010 and 2021, categorized (n = 37, each) as follows: group 1, MTC (men, 14; women, 23); group 2, PTC (men, 6; women, 31); group 3, FTC (men, 13; women, 24); or group 4, FTA (men, 10; women, 27). All patient data were acquired from our institutional database. Within the designated time period, there were 37 patients with MTCs, 290 with PTCs, 63 with FTCs, and 911 with FTAs. Equivalent patient samplings were achieved for groups 2–4 using a random number generator. Biographic data of all qualifying patients are presented in Table 1. Recruitment of study subjects is shown in Fig. 1.

**Table 1:** Age and sex distribution of the patients in the four subsets

	MTC	PTC	FTC	FTA	p
n	37	37	37	37	0.0512, ANOVA
mean age [years]	57	48	53	49	
SD	16	16	15	12	
Min	28	16	23	25	
Max	94	83	87	73	
males	14	6	13	10	< 0.0001, chi-square test
%	38	2	35	27	
females	23	31	24	27	
%	62	98	65	73	

MTC: medullary thyroid carcinoma, PTC: papillary thyroid carcinoma, FTC: follicular thyroid carcinoma, FTA: follicular thyroid adenoma, SD: standard deviation, MIN: minimum, MAX: maximum

Each enrollee underwent thyroidectomy in one of two surgical departments. All diagnoses of thyroid carcinoma or thyroid adenoma were confirmed histologically by board-certified pathologists with expertise in thyroid neoplasms.

In all patients, US devices used for preoperative examinations were equipped with high-resolution longitudinal probes transmitting at 10.0 MHz (LOGIQ P6 Pro; GE Healthcare, Chicago, IL, USA). The entire body of imaging data was stored in a picture archiving and communication system (PACS) for later analysis by two nuclear medicine specialists, each with > 10 years of experience reviewing > 2000 thyroid US examinations annually.

Patients were examined in supine positions, necks slightly extended. In doing so, both anterior and lateral neck areas were freely accessible by US probe. First, the entire right lobe was examined in transverse and longitudinal orientations. Then, the same procedure was applied step-wise to left lobe and to isthmus.

Focal thyroidal lesions were recorded in two dimensions and stored as above. Examiners assessed the following seven morphologic tumor criteria: (1) Volume (mL), calculated as v = 0.5 * (dx * dy * dz) using maximal lateral (dx), anteroposterior (dy), and craniocaudal (dz) axial diameters; (2) Shape, whether round (dx = dy = dz [± 10%]), oval (> 10% disparity in axial diameters, except TTW), irregular (undulating or complex shape), or TTW (anteroposterior diameter > lateral diameter, craniocaudal diameter discounted); (3) Contour (smooth vs. ill-defined); (4) Internal structure (homogeneous vs. non-homogeneous); (5) Echogenicity, whether hypoechogenic (below adjacent tissue level but not anechoic), hypoechogenic with cysts (anechoic components), hyperechogenic (beyond adjacent tissue level), or hyperechogenic with cysts; (6) Calcification (+/−); and (7) Focality (one or multiple sites).

Criteria (2) through (7) for focal lesions corresponded to those defined by Russ et al. [9].

### Artificial neural network architecture

This study was conducted to compare tumors by groups (i.e., MTC vs. PTC, MTC vs. FTC, MTC vs. FTA, PTC vs. FTC, PTC vs. FTA, and FTC vs. FTA) and with respect to the EU TI-RADS system, based on neural network processing of ultrasonographic traits. Only three hidden layers were involved, given the relative paucity of data. Demographic (age and sex) and morphologic characteristics (volume, shape, contour, structure, echogenicity, calcifications, and focality) served as input.

**Fig. 2:**
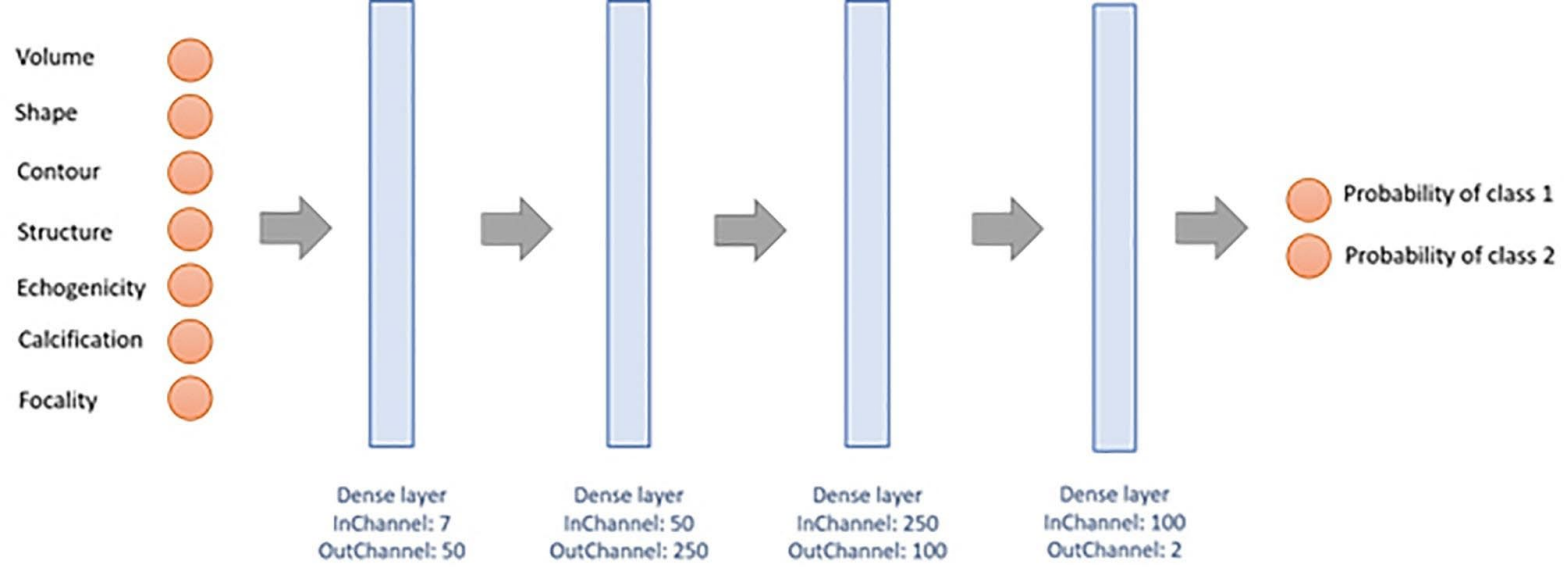
Architecture of the neural network with seven input neurons to discriminate among the classes of thyroid nodules

The network architecture is illustrated in Fig. 2. There are seven input neurons fully connected to three hidden layers of 50, 250, and 100 neurons respectively. Output is shown as a vector reflecting respective tumor probabilities. In the hidden layer, a rectified linear unit is invoked as activation function, the output layer assuming a sigmoidal function and the mean squared error a loss function. To evaluate the network, a leave-one-out cross-validation is carried out. Of all available datasets, 75% were used initially for training, 20% for validation, and 5% for testing. Features of the implemented artificial neural network are listed in Table 2. The neural network was implemented in Python, Tensorflow and Keras.

**Table 2:** Features of the implemented neural network

Layer	Shape	Number of Parameters	Activation Function
Input layer	(7,1)	0	-
Dense layer	[(7,50), (50)]	450	ReLu
Dense layer	[(7,250), (250)]	12,750	ReLu
Dense layer	[(7,100), (100)]	25,100	ReLu
Dense layer	[(7,2), (2)]	101	Sigmoid
Output	(2,1)	0	-

Shape: the numbers represent the data vectors, ReLU: Rectified linear unit

### Statistical analysis

Depending on the nature of data distribution, analysis of variance (ANOVA), Fisher’s exact test, or chi-square test was applied to assess differences among groups. Significance in linear relations was gauged via Pearson’s correlation coefficient, using multinomial logistic regression for multivariate relations. By default, confidence intervals of binary variables involved binomial distributions. In neural network performance analysis, the following metrics were generated: accuracy, sensitivity, specificity, positive predictive value, negative predictive value, Fleiss’ κ, Cohen’s κ, and F-score. All computations were driven by standard software (MATLAB vR2012b; The MathWorks Inc, Natick, MA, USA), setting significance at *p* < 0.05.

## Results

### Patient age and sex distributions

Mean patient age did not differ significantly (*p* = 0.0512, ANOVA), whereas female (vs. male) sex significantly predominated (*p* < 0.0001, chi-square test) across all four groups (see Table 1). The youngest (16 years) and the oldest (94 years) participants were within PTC and MTC groups, respectively, with male patients accounting for 2% and 38% therein.

### Ultrasonographic characteristics of categorized nodules

Average tumor volume determined by US differed significantly (*p* < 0.001, ANOVA). The smallest (0.01 mL) and the largest (122.67 mL) volumes recorded were within PTC and FTC groups, respectively (see Table 3). Equal numbers of subcentimeter nodules were found in the MTC and PTC groups (n = 7; 19%). Whereas in the FTC and FTA groups no subcentimeter nodules were detected.

**Table 3:** Tumor volume determined by ultrasound measurements in the four subsets

	MTC	PTC	FTC	FTA	p
mean [mL]	2.33	1.27	20.06	8.37	< 0.001, ANOVA
SD	3.07	1.71	26.00	9.90	
Min	0.06	0.01	0.48	0.29	
Max	12.39	9.67	122.67	44.20	

MTC: medullary thyroid carcinoma, PTC: papillary thyroid carcinoma, FTC: follicular thyroid carcinoma, FTA: follicular thyroid adenoma

**Fig. 3:**
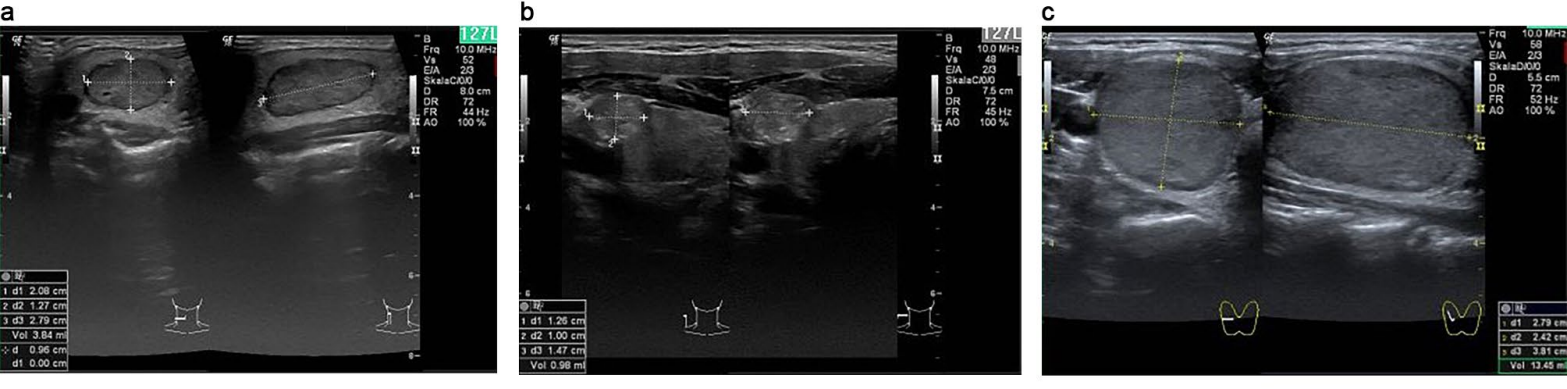
Sonographic appearances of follicular and parafollicular thyroid nodules. **a)** MTC right lobe, **b)** PTC right lobe, **c)** FTC right lobe. MTC: medullary thyroid carcinoma, PTC: papillary thyroid carcinoma, FTC: follicular thyroid carcinoma.

Specific US-based tumor characteristics (shape, contour, structure, echogenicity, calcifications, and focality) of the EU TI-RADS system were recorded for each lesion. Representative sonographic images of nodules are shown by group in Fig. 3, with distributions of features displayed presented in Table 4. In multivariate regression analysis, malignant nodules could be discriminated by US characteristics, such as ill-defined contour and calcifications (r = 0.46; *p* < 0.001). US-based discrimination between two entities is shown in Table 5. Regression analysis indicated that no single entity was distinguishable across all categorical groups (r = 0.18; *p* = 0.728) (see Table 5).

**Table 4:** Specific ultrasonographic tumor characteristics in the four subsets

	MTC	%	PTC	%	FTC	%	FTA	%	ANOVA
**shape**									0.951
round	9	24	10	27	6	16	6	16	
oval	17	46	18	49	26	70	25	68	
irregular	11	30	5	14	4	11	6	16	
ttw	0	0	4	11	1	3	0	0	
**contour**									0.025
smooth	12	32	18	49	17	46	25	68	
ill-defined	25	68	19	51	20	54	12	32	
**structure**									0.032
homogeneous	12	32	11	30	8	22	11	30	
inhomogeneous	25	68	26	70	29	78	14	38	
cystic	0	0	0	0	0	0	12	32	
**echogenicity**									0.0993
hypoechogeneous	33	89	29	78	26	70	23	62	
hyperechogeneous	4	11	5	14	8	22	14	38	
isoechogeneous	0	0	3	8	3	8	0	0	
**calcifications**									0.0002
abscent	17	46	20	54	29	78	32	86	
present	20	54	17	46	8	22	5	14	
**focality**									0.5622
unifocal	37	100	34	92	36	97	36	97	
multifdocal	0	0	3	8	1	3	1	3	

MTC: medullary thyroid carcinoma, PTC: papillary thyroid carcinoma, FTC: follicular thyroid carcinoma, FTA: follicular thyroid adenoma, ttw: taller-than-wide

**Table 5:** Multivariate regression analysis of ultrasound characteristics to discriminate between two subsets

	MTC		PTC		FTC		FTA	
	r	p	r	p	r	p	r	p
MTC	-	-	0.34	0.256	0.46	0.021	0.66	< 0.001
PTC			-	-	0.30	0.488	0.41	0.072
FTC					-	-	0.40	0.096
FTA							-	-

MTC: medullary thyroid carcinoma, PTC: papillary thyroid carcinoma, FTC: follicular thyroid carcinoma, FTA: follicular thyroid adenoma

**Fig. 4:**
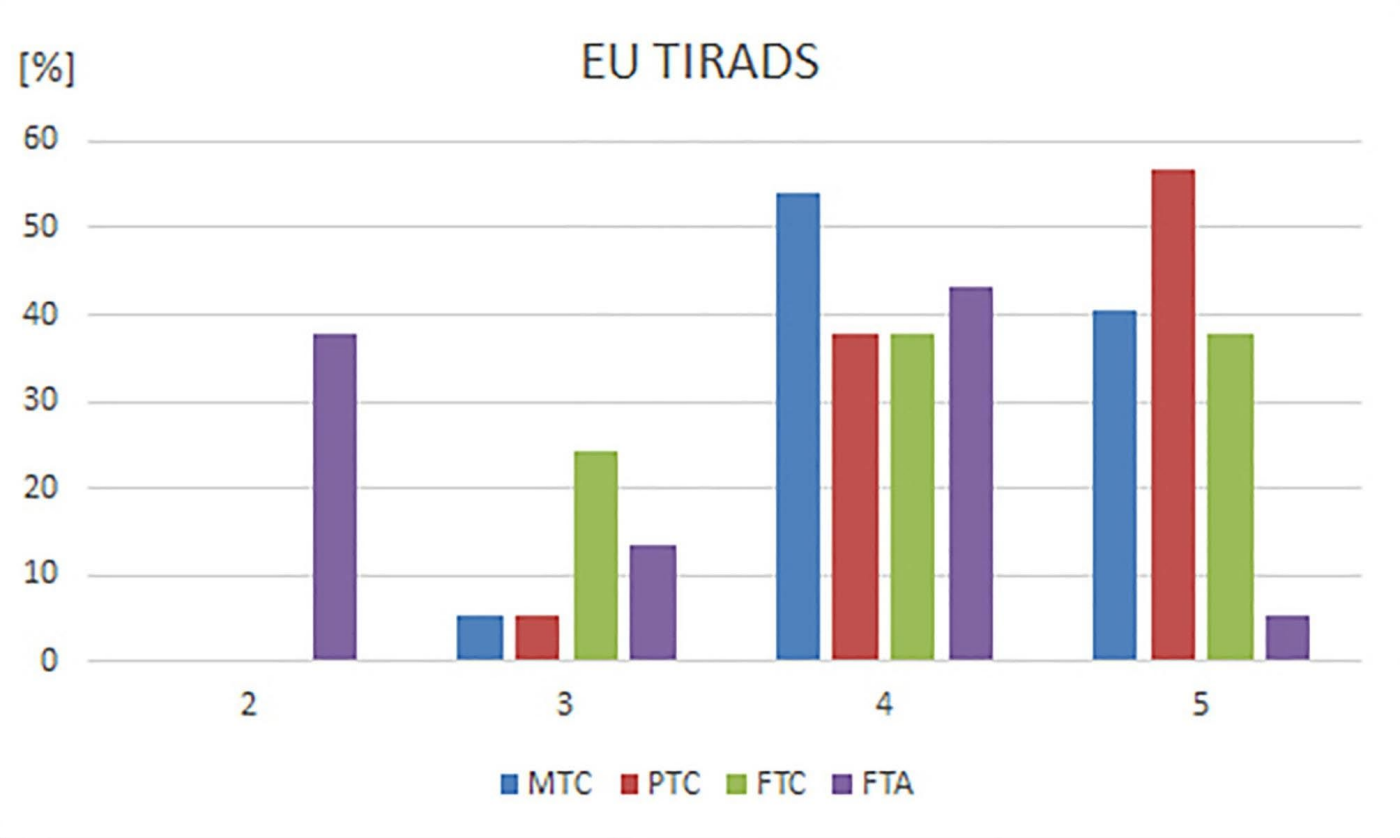
Distribution of the EU TIRADS classifications for subsets MTC, PTC, FTC and FTA. MTC: medullary thyroid carcinoma, PTC: papillary thyroid carcinoma, FTC: follicular thyroid carcinoma, FTA: follicular thyroid adenoma

Malignant nodules of MTC, PTC, and FTC groups were classified as EU TI-RADS 5 in 41%, 57%, and 38% of cases, respectively or as EU TI-RADS 4 in 54%, 38%, and 38% of cases, respectively. In the FTA group, 5% and 43% of nodules were classified as EU TI-RADS 5 or 4, respectively. EU TI-RADS classifications for all tumor groups are shown in Table 6 and in Fig. 4.

**Table 6:** EU TIRADS classification for nodules in the four subsets

	EU TIRADS 2	%	EU TIRADS 3	%	EU TIRADS 4	%	EU TIRADS 5	%
MTC	0	0	2	5	20	54	15	41
PTC	0	0	2	5	14	38	21	57
FTC	0	0	9	24	14	38	14	38
FTA	14	38	5	14	16	43	2	5

MTC: medullary thyroid carcinoma, PTC: papillary thyroid carcinoma, FTC: follicular thyroid carcinoma, FTA: follicular thyroid adenoma

### Artificial neural network performance

To determine how the artificial neural network performed, we calculated sensitivity, specificity, and accuracy on a pair-wise basis. Sensitivity, specificity, and accuracy were lowest for MTC vs. PTC (0.65, 0.57, and 0.59, respectively) and highest for MTC vs. FTA (0.97, 0.98, and 0.98, respectively) (see Table 7).

**Table 7:** Performance data of the neural network

	Sensitivity	Specificity		Accuracy	CI 95%		PPV	NPV
MTC vs. PTC	0.65	0.57		0.59	0.57–0.61		0.41	0.78
MTC vs. FTC	0.90	0.95		0.93	0.91–0.95		0.95	0.89
MTC vs. FTA	0.97	0.98		0.98	0.96–0.99		0.99	0.97
PTC vs. FTC	0.90	0.97		0.94	0.92–0.96		0.97	0.89
PTC vs. FTA	0.96	0.98		0.97	0.95–0.99		0.98	0.96
FTC vs. FTA	0.77	0.65		0.69	0.67–0.71		0.54	0.84
MTC + PTC + FTC vs. FTA	0.70	0.94		0.83	0.81–0.85		0.97	0.90

MTC: medullary thyroid carcinoma, PTC: papillary thyroid carcinoma, FTC: follicular thyroid carcinoma, FTA: follicular thyroid adenoma

In discriminating benign or low-risk nodules (EU TI-RADS 2 or 3) from intermediate or high-risk nodules (EU TI-RADS 4 or 5), the neural network achieved an agreement of 97%, 92%, 19%, and 95% for MTC, PTC, FTC, and FTA groups. Corresponding values generated by the trained examiner were 95%, 95%, 76%, and 51%, respectively.

Overall, EU TI-RADS classifications of 2, 3, 4, and 5 were correctly assigned by the neural network to MTC, PTC, FTC, and FTA groups in 54%, 51%, 18%, and 68% of cases.

## Discussion

This retrospective study was undertaken to define US characteristics of histologically confirmed follicular and parafollicular thyroid neoplasms. We tested the hypothesis that US features of thyroid cancers (medullary, papillary, or follicular) and adenomas are distinctive, implementing an artificial neural network analysis for proof of concept.

In most cases biopsy allows to differentiate between benign and malignant nodules. However, in some cases further work-up is undispensable. Due to inadequate samplings, inaccessability for FNAB or patient´s refusal some nodules remain unclassified. For these nodules ultrasonography classification systems (such as TIRADS) help to differentiate between a malignant and benign nature. With respect to potentially malignant nodules we consider it reasonable to further assess the tumor entity. This assessment of the tumor entity may contribute to define the most appropriate approach (surgical treatment versus active surveillance). In our oppinion the future clinical significance of artificial neural network analysis may lie in differentiating those nodules which remain unclassified by FNAB.

Data on US properties of thyroid malignancies abound in past reports, including one recent review of 52 observational studies aimed at US imaging of malignant thyroid nodules [12]. However, the non-uniformity of methodologies applied within this compilation and a failure to correlate US characteristics with specific histotypes of thyroid cancer are striking limitations. US characteristics of benign thyroid nodules have likewise been described and compared with those of malignant lesions [13, 14], but benign nodules in these studies were confirmed solely by biopsies and could not be profiled histologically.

In our investigation, we used the same US methodology for all patients examined. Nodules were eligible only if histologic classifications were available. Our results were also based on equivalent patient numbers to avoid sampling bias, and we used these particular cases for training of the artificial neural network.

All benign nodules were histologically confirmed as follicular adenomas. Because follicular adenomas and follicular carcinomas are alike in macroscopic architecture, differentiation by way of US may be problematic [4, 15]. Indeed, US characteristics of FTCs and FTAs were similar in our hands. Yet, the same was true of a large, recently reported meta-analysis [15], where the most reliable (though often absent) means of distinguishing FTCs from FTAs was tumor protrusion. On the other hand, we determined that FTCs surpassed FTAs in mean tumor volume, a finding substantiated by others [16–18]. FTCs were also classifiable by EU TI-RADS in 24% of cases in our series. This was the highest value among groupings of malignant lesions, and the neural network was able to discriminate between FTCs and FTAs with moderate accuracy (0.69).

US characteristics of MTCs and PTCs were largely identical, too. With exception of TTW configuration (confined to PTCs), the vast majority of MTCs and PTCs appeared hypoechogenic, demonstrating calcifications in ~ 50% of cases. Consequently, the neural network was vastly less accurate in MTC vs. PTC (0.59) than in MTC vs. FTA (0.98) determinations. The findings of Liu [19] seem to corroborate the observed link between TTW shape and PTCs, confirming significantly more PTCs (vs. MTCs) with TTW shapes. Calcifications were detectable in ~ 50% of each group, but MTCs were larger than PTCs. Only tumor diameters were provided, thus prohibiting a direct comparison of mean tumor volumes.

In the present study, PTCs and FTAs again shared many US characteristics. Ill-defined contours were identified in both (PTCs, 51%; FTAs, 32%), as were calcifications (PTCs, 46%; FTAs, 14%). Only TTW shape was reliably diagnostic of PTC. In comparing US characteristics of PTCs and benign nodules, Fang et al. [3] detected ill-defined margins and calcifications in significant proportions of PTCs and benign lesions, and there were benign lesions with TTW shapes. However, this was a heterogeneous group comprised of nodular goiters, adenomas, and nodules in Hashimoto´s thyroiditis.

As presently tested, the power of our artificial neural network to discriminate among thyroid nodules through US characteristics seemed dependent on histologic features. Although distinguishing MTC from FTA was correct in most instances, less success was afforded for MTC vs. PTC. Because MTCs and PTCs frequently share US characteristics, this implies some degree of macroscopic consistency. The network’s capacity to correctly discern benign and low-risk nodules from those of intermediate or high risk was similarly a function of histology. For MTCs, the discrimination rate was fairly high, whereas FTAs showed the lowest rate. This possibly explains the rather high classification rate for EU TI-RADS 4/5 in benign nodules.

Untile today, most AI applications in thyroid disease have focused on estimation of the malignancy risk of nodules [20]. In a retrospective study of follicular thyroid nodules, Xu et al. achieved an accuracy of 0.71 for discriminating between benign and malignant lesions by neural network analysis [21]. Hence, the potential benefit of this approach in helping radiologists separate FTCs from nodules poorly distinguishable by US was duly illustrated. Recently, a large meta-analysis examined the diagnostic utility of CNN analytics in classifying indeterminate thyroid nodules as benign or malignant through US imaging [22]. This undertaking encompassed 75 studies of > 46,000 nodules. Ultimately, CNN (sensitivity, 0.85; specificity, 0.82) and radiomic (sensitivity, 0.87; specificity, 0.84) analyses compared favorably, underscoring the high-performance capability of machine learning procedures in classifying thyroid nodules.

There are inherent limitations of this study to acknowledge. Its retrospective and single-center design as well as the limited number of patients perhaps diminished the statistical power of our results. We included only those thyroid tumors from our database that were clearly encoded, and only surgically treated patients at our facility with available pathologic reports were considered. For image segmentation we used a visual approach. This process can be very subjective and prone to inter and intra-observer variations. However, an observer adherent bias may have reduced the performance of our nodule classification by artificial neural network analysis. Furthermore, the various protocols applied were clinically based and non-standardized, and the small number of patients involved (not perfectly matched) may have introduced significant outcome bias. The major limitation of our study might be that the number of test data was small. Therefore, we recommend the training of the neural network for future studies with larger data sets. Finally, we used a concise rather than comprehensive neural network model, requiring some simplification of output functions.

## Conclusions

Our data indicate that some MTCs, PTCs, FTCs, and FTAs have distinctive sonographic characteristics. However, a significant overlap of these characteristics may impede an explicit classification. Further prospective investigations involving larger patient and nodule numbers and multicenter access should be pursued to determine if neural networks of this sort are beneficial, helping to classify neoplasms of the thyroid gland.

## Data Availability

Available.
